# 10-(4-Methyl­benzyl­idene)anthracen-9(10*H*)-one

**DOI:** 10.1107/S1600536812000827

**Published:** 2012-01-21

**Authors:** Abdulrahman I. Almansour, Natarajan Arumugam, Usama Karama, Ibrahim Abdul Razak, Suhana Arshad

**Affiliations:** a Department of Chemistry, College of Sciences, King Saud University, PO Box 2455, Riyadh 11451, Saudi Arabia; b School of Physics, Universiti Sains Malaysia, 11800 USM, Penang, Malaysia

## Abstract

In the title compound, C_22_H_16_O, the six-membered ring within the anthrone moiety adopts a shallow boat conformation, with puckering parameters *Q* = 0.2860 (17) Å, Θ = 99.1 (3)° and Φ = 114.8 (3)°. The dihedral angle between the outer benzene rings is 26.53 (8)°. The mean plane through the anthrone ring system makes a dihedral angle of 38.73 (6)° with the pendant benzene ring. In the crystal, mol­ecules are linked by C—H⋯O hydrogen bonds into zigzag chains propagating along the *c*-axis direction and weak C—H⋯π inter­actions further consolidate the structure.

## Related literature

For a related structure and background to anthrone derivatives, see: Arumugam *et al.* (2011 ▶). For related structures, see: Wen & Li (2008 ▶); Zhou *et al.* (2004 ▶). For the synthesis, see: Prinz *et al.* (2003 ▶). For ring conformations, see: Cremer & Pople (1975 ▶). For the stability of the temperature controller used in the data collection, see: Cosier & Glazer (1986 ▶).
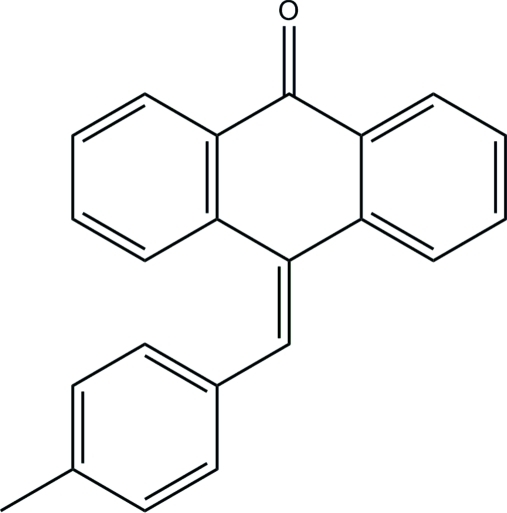


## Experimental

### 

#### Crystal data

C_22_H_16_O
*M* *_r_* = 296.35
Orthorhombic, 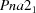

*a* = 7.2959 (1) Å
*b* = 16.3853 (2) Å
*c* = 13.0028 (2) Å
*V* = 1554.43 (4) Å^3^
*Z* = 4
Mo *K*α radiation
μ = 0.08 mm^−1^
*T* = 100 K
0.33 × 0.27 × 0.21 mm

#### Data collection

Bruker SMART APEXII CCD diffractometer
Absorption correction: multi-scan (*SADABS*; Bruker, 2009 ▶) *T*_min_ = 0.975, *T*_max_ = 0.985
20184 measured reflections
2757 independent reflections
2575 reflections with *I* > 2σ(*I*)
*R*_int_ = 0.030

#### Refinement

*R*[*F* ^2^ > 2σ(*F* ^2^)] = 0.043
*wR*(*F* ^2^) = 0.122
*S* = 1.09
2757 reflections
209 parameters
1 restraint
H-atom parameters constrained
Δρ_max_ = 0.40 e Å^−3^
Δρ_min_ = −0.19 e Å^−3^
Absolute structure: Flack (1983 ▶), 2319 Friedel pairs
Flack parameter: 0 (10)

### 

Data collection: *APEX2* (Bruker, 2009 ▶); cell refinement: *SAINT* (Bruker, 2009 ▶); data reduction: *SAINT*; program(s) used to solve structure: *SHELXTL* (Sheldrick, 2008 ▶); program(s) used to refine structure: *SHELXTL*; molecular graphics: *SHELXTL*; software used to prepare material for publication: *SHELXTL* and *PLATON* (Spek, 2009 ▶).

## Supplementary Material

Crystal structure: contains datablock(s) global, I. DOI: 10.1107/S1600536812000827/hb6592sup1.cif

Structure factors: contains datablock(s) I. DOI: 10.1107/S1600536812000827/hb6592Isup2.hkl

Supplementary material file. DOI: 10.1107/S1600536812000827/hb6592Isup3.cml

Additional supplementary materials:  crystallographic information; 3D view; checkCIF report

## Figures and Tables

**Table 1:** Hydrogen-bond geometry (Å, °) *Cg*1 and *Cg*2 are the centroids of the C1–C6 and C16–C21 rings, respectively.

*D*—H⋯*A*	*D*—H	H⋯*A*	*D*⋯*A*	*D*—H⋯*A*
C3—H3*A*⋯O1^i^	0.95	2.35	3.275 (2)	164
C22—H22*C*⋯*Cg*1^ii^	0.98	2.94	3.726 (2)	138
C17—H17*A*⋯*Cg*2^iii^	0.95	2.76	3.5073 (16)	136

Symmetry codes: (i) 
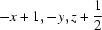
; (ii) 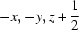; (iii) 
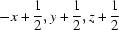
.

**Fig. 1.**
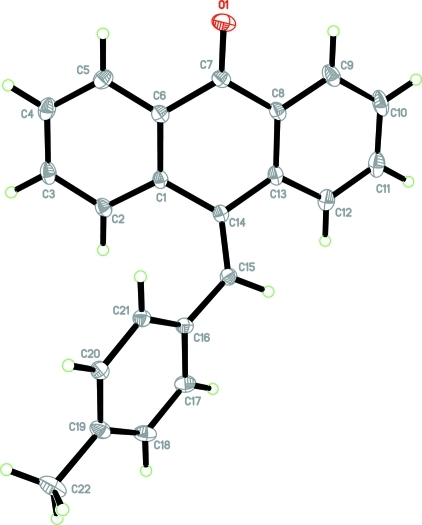
The molecular structure of the title compound, showing 30% probability displacement ellipsoids.

**Fig. 2.**
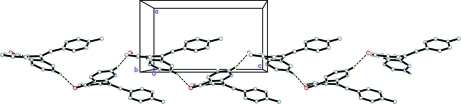
The crystal packing of the title compound. The H atoms not involved in the intermolecular interactions (dashed lines) have been omitted for clarity.

## supplementary crystallographic information

### Comment

As part of our ongoing studies of anthrone derivatives (Arumugam *et al.*, 2011), we have undertaken the X-ray crystal structure determination of the title compound, (I).

In the molecular structure (Fig 1), the six-membered ring (C1/C6–C8/C13/C14) within the anthracene moiety adopts a boat conformation with puckering parameters Q= 0.2860 (17) Å, Θ= 99.1 (3)° and Φ= 114.8 (3)° (Cremer & Pople, 1975). This differs from the planar anthracene ring reported in related structure (Arumugam *et al.*, 2011). The mean plane through the anthracene ring (C1–C14) makes a dihedral angle of 38.73 (6)° with the phenyl ring (C16–C21). The bond lengths and angles are comparable those in the related structure (Wen & Li, 2008; Zhou *et al.*, 2004).

The crystal packing is shown in Fig. 2. The molecules are linked by the intermolecular C3—H3A···O1 hydrogen bonds (Table 1) into infinite one dimensional zigzag chain along the *c*-axis. In addition, the crystal structure are further stabilized by the intermolecular C22—H22C···*Cg1* and C17—H17A···*Cg2* (Table 1) interactions (*Cg*1 and *Cg*2 are the centroids of C1–C6 and C16–C21 rings, respectively).

### Experimental

The title compound was synthesized as reported (Prinz *et al.*, 2003) and crystallized EtOAc by slow evaporation technique.

### Refinement

All H atoms were positioned geometrically [C–H = 0.95 and 0.98 Å] and refined using a riding model with *U*_iso_(H) = 1.2 and 1.5 *U*_eq_(C). A rotating group model was applied to the methyl groups. A total of 2319 Freidel pairs were used to determine the absolute structure.

### Crystal data

**Table:**

C_22_H_16_O	*F*(000) = 624
*M**_r_* = 296.35	*D*_x_ = 1.266 Mg m^−^^3^
Orthorhombic, *P**n**a*2_1_	Mo *K*α radiation, λ = 0.71073 Å
Hall symbol: P 2c -2n	Cell parameters from 8371 reflections
*a* = 7.2959 (1) Å	θ = 3.1–31.7°
*b* = 16.3853 (2) Å	µ = 0.08 mm^−^^1^
*c* = 13.0028 (2) Å	*T* = 100 K
*V* = 1554.43 (4) Å^3^	Block, yellow
*Z* = 4	0.33 × 0.27 × 0.21 mm

### Data collection

**Table:**

Bruker SMART APEXII CCD diffractometer	2757 independent reflections
Radiation source: fine-focus sealed tube	2575 reflections with *I* > 2σ(*I*)
graphite	*R*_int_ = 0.030
φ and ω scans	θ_max_ = 31.8°, θ_min_ = 2.0°
Absorption correction: multi-scan (*SADABS*; Bruker, 2009)	*h* = −10→10
*T*_min_ = 0.975, *T*_max_ = 0.985	*k* = −24→21
20184 measured reflections	*l* = −19→17

### Refinement

**Table:**

Refinement on *F*^2^	Secondary atom site location: difference Fourier map
Least-squares matrix: full	Hydrogen site location: inferred from neighbouring sites
*R*[*F*^2^ > 2σ(*F*^2^)] = 0.043	H-atom parameters constrained
*wR*(*F*^2^) = 0.122	*w* = 1/[σ^2^(*F*_o_^2^) + (0.0831*P*)^2^ + 0.1277*P*] where *P* = (*F*_o_^2^ + 2*F*_c_^2^)/3
*S* = 1.09	(Δ/σ)_max_ = 0.002
2757 reflections	Δρ_max_ = 0.40 e Å^−^^3^
209 parameters	Δρ_min_ = −0.19 e Å^−^^3^
1 restraint	Absolute structure: Flack (1983), 2319 Friedel pairs
Primary atom site location: structure-invariant direct methods	Flack parameter: 0 (10)

### Special details

**Table:**

Experimental. The crystal was placed in the cold stream of an Oxford Cryosystems Cobra open-flow nitrogen cryostat (Cosier & Glazer, 1986) operating at 100.0 (1) K.
Geometry. All e.s.d.'s (except the e.s.d. in the dihedral angle between two l.s. planes) are estimated using the full covariance matrix. The cell e.s.d.'s are taken into account individually in the estimation of e.s.d.'s in distances, angles and torsion angles; correlations between e.s.d.'s in cell parameters are only used when they are defined by crystal symmetry. An approximate (isotropic) treatment of cell e.s.d.'s is used for estimating e.s.d.'s involving l.s. planes.
Refinement. Refinement of *F*^2^ against ALL reflections. The weighted *R*-factor *wR* and goodness of fit *S* are based on *F*^2^, conventional *R*-factors *R* are based on *F*, with *F* set to zero for negative *F*^2^. The threshold expression of *F*^2^ > σ(*F*^2^) is used only for calculating *R*-factors(gt) *etc*. and is not relevant to the choice of reflections for refinement. *R*-factors based on *F*^2^ are statistically about twice as large as those based on *F*, and *R*- factors based on ALL data will be even larger.

### Fractional atomic coordinates and isotropic or equivalent isotropic displacement parameters (Å^2^)

**Table:**

	*x*	*y*	*z*	*U*_iso_*/*U*_eq_	
O1	0.22961 (18)	0.09172 (9)	0.36336 (11)	0.0332 (3)	
C1	0.34392 (19)	0.11506 (9)	0.63231 (12)	0.0204 (3)	
C2	0.4267 (2)	0.07120 (10)	0.71248 (13)	0.0249 (3)	
H2A	0.4357	0.0949	0.7789	0.030*	
C3	0.4956 (2)	−0.00689 (11)	0.69531 (15)	0.0297 (4)	
H3A	0.5527	−0.0357	0.7500	0.036*	
C4	0.4818 (2)	−0.04321 (11)	0.59886 (16)	0.0311 (4)	
H4A	0.5270	−0.0969	0.5880	0.037*	
C5	0.4016 (2)	−0.00049 (10)	0.51897 (15)	0.0274 (3)	
H5A	0.3917	−0.0250	0.4530	0.033*	
C6	0.3352 (2)	0.07847 (10)	0.53448 (12)	0.0223 (3)	
C7	0.2696 (2)	0.12531 (10)	0.44437 (12)	0.0234 (3)	
C8	0.2672 (2)	0.21516 (11)	0.45515 (12)	0.0239 (3)	
C9	0.2547 (3)	0.26344 (13)	0.36613 (16)	0.0328 (4)	
H9A	0.2415	0.2381	0.3008	0.039*	
C10	0.2613 (3)	0.34737 (13)	0.37306 (18)	0.0384 (5)	
H10A	0.2514	0.3799	0.3129	0.046*	
C11	0.2828 (2)	0.38405 (12)	0.46872 (18)	0.0351 (4)	
H11A	0.2905	0.4418	0.4733	0.042*	
C12	0.2931 (2)	0.33765 (10)	0.55740 (16)	0.0281 (3)	
H12A	0.3071	0.3638	0.6221	0.034*	
C13	0.2829 (2)	0.25202 (10)	0.55238 (13)	0.0227 (3)	
C14	0.2792 (2)	0.20019 (9)	0.64518 (12)	0.0203 (3)	
C15	0.2111 (2)	0.23176 (9)	0.73396 (12)	0.0223 (3)	
H15A	0.1904	0.2890	0.7325	0.027*	
C16	0.1643 (2)	0.19219 (9)	0.83166 (12)	0.0220 (3)	
C17	0.2006 (2)	0.23303 (10)	0.92404 (14)	0.0274 (3)	
H17A	0.2577	0.2851	0.9224	0.033*	
C18	0.1542 (2)	0.19832 (12)	1.01808 (13)	0.0293 (3)	
H18A	0.1828	0.2265	1.0799	0.035*	
C19	0.0662 (2)	0.12249 (11)	1.02327 (13)	0.0269 (3)	
C20	0.0247 (2)	0.08341 (10)	0.93057 (13)	0.0240 (3)	
H20A	−0.0385	0.0327	0.9321	0.029*	
C21	0.0736 (2)	0.11680 (9)	0.83647 (12)	0.0223 (3)	
H21A	0.0454	0.0884	0.7748	0.027*	
C22	0.0189 (3)	0.08462 (15)	1.12512 (15)	0.0386 (4)	
H22A	0.0738	0.1168	1.1807	0.058*	
H22B	0.0665	0.0287	1.1276	0.058*	
H22C	−0.1146	0.0836	1.1335	0.058*	

### Atomic displacement parameters (Å^2^)

**Table:**

	*U*^11^	*U*^22^	*U*^33^	*U*^12^	*U*^13^	*U*^23^
O1	0.0306 (6)	0.0475 (7)	0.0215 (5)	0.0048 (5)	−0.0009 (5)	−0.0047 (5)
C1	0.0160 (6)	0.0216 (6)	0.0235 (6)	0.0018 (4)	0.0013 (5)	0.0038 (5)
C2	0.0206 (6)	0.0296 (7)	0.0245 (6)	0.0027 (6)	−0.0003 (6)	0.0064 (6)
C3	0.0224 (6)	0.0301 (7)	0.0364 (9)	0.0063 (6)	0.0030 (6)	0.0117 (7)
C4	0.0247 (7)	0.0260 (7)	0.0425 (9)	0.0062 (6)	0.0062 (7)	0.0039 (7)
C5	0.0226 (7)	0.0275 (7)	0.0321 (8)	0.0018 (6)	0.0051 (6)	−0.0029 (6)
C6	0.0172 (6)	0.0272 (7)	0.0225 (6)	0.0009 (5)	0.0019 (5)	0.0028 (5)
C7	0.0178 (6)	0.0317 (7)	0.0207 (7)	0.0026 (5)	0.0025 (5)	0.0010 (6)
C8	0.0181 (6)	0.0317 (7)	0.0219 (7)	0.0035 (5)	0.0034 (5)	0.0074 (6)
C9	0.0257 (7)	0.0466 (10)	0.0261 (7)	0.0071 (7)	0.0057 (6)	0.0138 (8)
C10	0.0300 (8)	0.0449 (10)	0.0403 (10)	0.0085 (7)	0.0091 (7)	0.0259 (9)
C11	0.0244 (8)	0.0312 (8)	0.0495 (11)	0.0028 (6)	0.0096 (7)	0.0173 (8)
C12	0.0205 (7)	0.0250 (7)	0.0387 (9)	−0.0004 (5)	0.0062 (6)	0.0080 (7)
C13	0.0164 (6)	0.0252 (7)	0.0264 (7)	0.0012 (5)	0.0019 (5)	0.0071 (6)
C14	0.0168 (6)	0.0221 (6)	0.0221 (6)	−0.0005 (4)	−0.0005 (5)	0.0030 (5)
C15	0.0225 (7)	0.0215 (6)	0.0227 (7)	−0.0007 (5)	0.0008 (5)	0.0006 (5)
C16	0.0219 (7)	0.0242 (6)	0.0198 (6)	0.0007 (5)	0.0010 (6)	−0.0011 (5)
C17	0.0260 (7)	0.0315 (8)	0.0249 (7)	−0.0038 (6)	0.0009 (6)	−0.0060 (6)
C18	0.0264 (8)	0.0416 (9)	0.0198 (7)	−0.0010 (6)	−0.0008 (6)	−0.0062 (6)
C19	0.0205 (7)	0.0394 (8)	0.0208 (6)	0.0018 (6)	0.0013 (6)	0.0031 (6)
C20	0.0208 (6)	0.0272 (7)	0.0239 (7)	0.0019 (5)	0.0011 (6)	0.0025 (6)
C21	0.0224 (6)	0.0235 (6)	0.0210 (6)	0.0001 (5)	0.0020 (5)	−0.0002 (5)
C22	0.0322 (9)	0.0613 (12)	0.0224 (8)	−0.0067 (8)	0.0017 (7)	0.0081 (8)

### Geometric parameters (Å, °)

**Table:**

O1—C7	1.224 (2)	C11—H11A	0.9500
C1—C2	1.403 (2)	C12—C13	1.407 (2)
C1—C6	1.408 (2)	C12—H12A	0.9500
C1—C14	1.482 (2)	C13—C14	1.476 (2)
C2—C3	1.393 (2)	C14—C15	1.359 (2)
C2—H2A	0.9500	C15—C16	1.467 (2)
C3—C4	1.392 (3)	C15—H15A	0.9500
C3—H3A	0.9500	C16—C17	1.400 (2)
C4—C5	1.382 (3)	C16—C21	1.403 (2)
C4—H4A	0.9500	C17—C18	1.390 (2)
C5—C6	1.396 (2)	C17—H17A	0.9500
C5—H5A	0.9500	C18—C19	1.400 (3)
C6—C7	1.480 (2)	C18—H18A	0.9500
C7—C8	1.479 (2)	C19—C20	1.398 (2)
C8—C13	1.406 (2)	C19—C22	1.503 (3)
C8—C9	1.405 (2)	C20—C21	1.387 (2)
C9—C10	1.379 (3)	C20—H20A	0.9500
C9—H9A	0.9500	C21—H21A	0.9500
C10—C11	1.390 (4)	C22—H22A	0.9800
C10—H10A	0.9500	C22—H22B	0.9800
C11—C12	1.383 (3)	C22—H22C	0.9800
			
C2—C1—C6	118.21 (14)	C13—C12—H12A	119.8
C2—C1—C14	122.37 (15)	C8—C13—C12	118.33 (15)
C6—C1—C14	119.25 (14)	C8—C13—C14	119.12 (14)
C3—C2—C1	120.46 (16)	C12—C13—C14	122.48 (15)
C3—C2—H2A	119.8	C15—C14—C13	118.82 (13)
C1—C2—H2A	119.8	C15—C14—C1	124.79 (14)
C4—C3—C2	120.73 (15)	C13—C14—C1	116.33 (14)
C4—C3—H3A	119.6	C14—C15—C16	130.74 (13)
C2—C3—H3A	119.6	C14—C15—H15A	114.6
C5—C4—C3	119.43 (15)	C16—C15—H15A	114.6
C5—C4—H4A	120.3	C17—C16—C21	118.16 (14)
C3—C4—H4A	120.3	C17—C16—C15	119.18 (13)
C4—C5—C6	120.50 (17)	C21—C16—C15	122.54 (14)
C4—C5—H5A	119.8	C18—C17—C16	120.85 (15)
C6—C5—H5A	119.8	C18—C17—H17A	119.6
C5—C6—C1	120.63 (15)	C16—C17—H17A	119.6
C5—C6—C7	118.58 (15)	C17—C18—C19	121.12 (15)
C1—C6—C7	120.60 (14)	C17—C18—H18A	119.4
O1—C7—C8	121.76 (15)	C19—C18—H18A	119.4
O1—C7—C6	121.69 (15)	C20—C19—C18	117.66 (15)
C8—C7—C6	116.42 (14)	C20—C19—C22	121.40 (16)
C13—C8—C9	120.29 (16)	C18—C19—C22	120.94 (16)
C13—C8—C7	120.79 (13)	C21—C20—C19	121.62 (14)
C9—C8—C7	118.89 (16)	C21—C20—H20A	119.2
C10—C9—C8	120.4 (2)	C19—C20—H20A	119.2
C10—C9—H9A	119.8	C20—C21—C16	120.53 (15)
C8—C9—H9A	119.8	C20—C21—H21A	119.7
C9—C10—C11	119.57 (17)	C16—C21—H21A	119.7
C9—C10—H10A	120.2	C19—C22—H22A	109.5
C11—C10—H10A	120.2	C19—C22—H22B	109.5
C12—C11—C10	120.96 (17)	H22A—C22—H22B	109.5
C12—C11—H11A	119.5	C19—C22—H22C	109.5
C10—C11—H11A	119.5	H22A—C22—H22C	109.5
C11—C12—C13	120.45 (18)	H22B—C22—H22C	109.5
C11—C12—H12A	119.8		
			
C6—C1—C2—C3	−0.9 (2)	C9—C8—C13—C14	174.59 (15)
C14—C1—C2—C3	−176.04 (15)	C7—C8—C13—C14	−7.4 (2)
C1—C2—C3—C4	−0.8 (3)	C11—C12—C13—C8	1.7 (2)
C2—C3—C4—C5	1.2 (3)	C11—C12—C13—C14	−175.30 (15)
C3—C4—C5—C6	0.1 (3)	C8—C13—C14—C15	−150.02 (15)
C4—C5—C6—C1	−1.8 (2)	C12—C13—C14—C15	27.0 (2)
C4—C5—C6—C7	173.23 (15)	C8—C13—C14—C1	27.30 (19)
C2—C1—C6—C5	2.2 (2)	C12—C13—C14—C1	−155.71 (14)
C14—C1—C6—C5	177.49 (14)	C2—C1—C14—C15	−32.6 (2)
C2—C1—C6—C7	−172.75 (14)	C6—C1—C14—C15	152.29 (16)
C14—C1—C6—C7	2.6 (2)	C2—C1—C14—C13	150.25 (14)
C5—C6—C7—O1	18.3 (2)	C6—C1—C14—C13	−24.9 (2)
C1—C6—C7—O1	−166.66 (15)	C13—C14—C15—C16	169.27 (16)
C5—C6—C7—C8	−157.66 (14)	C1—C14—C15—C16	−7.8 (3)
C1—C6—C7—C8	17.4 (2)	C14—C15—C16—C17	142.24 (19)
O1—C7—C8—C13	169.11 (15)	C14—C15—C16—C21	−41.8 (3)
C6—C7—C8—C13	−14.9 (2)	C21—C16—C17—C18	2.4 (2)
O1—C7—C8—C9	−12.8 (2)	C15—C16—C17—C18	178.48 (17)
C6—C7—C8—C9	163.10 (15)	C16—C17—C18—C19	−1.5 (3)
C13—C8—C9—C10	1.3 (3)	C17—C18—C19—C20	−0.7 (3)
C7—C8—C9—C10	−176.73 (16)	C17—C18—C19—C22	179.07 (18)
C8—C9—C10—C11	0.8 (3)	C18—C19—C20—C21	2.0 (2)
C9—C10—C11—C12	−1.6 (3)	C22—C19—C20—C21	−177.81 (17)
C10—C11—C12—C13	0.3 (3)	C19—C20—C21—C16	−1.1 (2)
C9—C8—C13—C12	−2.5 (2)	C17—C16—C21—C20	−1.1 (2)
C7—C8—C13—C12	175.48 (14)	C15—C16—C21—C20	−177.09 (14)

### Hydrogen-bond geometry (Å, °)

**Table:**

Cg1 and Cg2 are the centroids of the C1–C6 and C16–C21 rings, respectively.

**Table:**

*D*—H···*A*	*D*—H	H···*A*	*D*···*A*	*D*—H···*A*
C3—H3A···O1^i^	0.95	2.35	3.275 (2)	164
C22—H22C···Cg1^ii^	0.98	2.94	3.726 (2)	138
C17—H17A···Cg2^iii^	0.95	2.76	3.5073 (16)	136

Symmetry codes: (i) −*x*+1, −*y*, *z*+1/2; (ii) −*x*, −*y*, *z*+1/2; (iii) −*x*+1/2, *y*+1/2, *z*+1/2.
